# Synthesis and crystal structure of bis­(9-mesityl-9,10-di­hydro-10-aza-9-borabenzo[*h*]quinolinato-κ^2^ *N* ^1^,*N* ^10^)zinc(II)

**DOI:** 10.1107/S2056989023009192

**Published:** 2023-10-24

**Authors:** Yannik Appiarius, Pim Puylaert, Anne Staubitz

**Affiliations:** a University of Bremen, Institute for Organic and Analytical Chemistry, 28359 Bremen, Germany; b University of Bremen, MAPEX Center for Materials and Processes, 28359 Bremen, Germany; c University of Bremen, Institute for Inorganic Chemistry and Crystallography, 28359 Bremen, Germany; Universidad de Los Andes Mérida, Venezuela

**Keywords:** crystal structure, 1,2-aza­borinine, boron-nitro­gen, zinc, bidentate ligand, Hirshfeld analysis

## Abstract

The title compound, [Zn(C_20_H_18_BN_2_)_2_] (**Zn** ***L*** **_2_**), is an overall uncharged chelate that consists of two units of an N*H*-deprotonated 10-aza-9-borabenzo[*h*]quinoline ligand (*L*) per Zn^II^ center. It was synthesized in two steps by treating the protonated ligand **HL** with lithium bis­(tri­methyl­sil­yl)amide and further conversion with di­ethyl­zinc. Its asymmetric unit comprises one **ZnL** fragment; the mol­ecule is completed by application of inversion symmetry at Zn. Due to the fourfold coordination with nitro­gen atoms, the zinc(II) ion is located in a distorted tetra­hedral environment. Besides the relatively short N—Zn bonds, **Zn** ***L*** **_2_** is characterized by the significant protrusion of the central ion from the plane of the ligand backbone. The crystal structure is consolidated by intra- and inter­molecular π–π stacking inter­actions, while the polarized B—N bond is barely involved in any close atom contacts.

## Abstract 2

The N*H*-deprotonation of a 10-aza-9-borabenzo[*h*]quinoline yields a bidentate ligand that was used for the synthesis of a 2:1 coordination complex with zinc. Its crystal packing is dominated by intense intra- and inter­molecular π–π stacking inter­actions.

## 1. Chemical context

1,2-Aza­borinine is an aromatic six-membered ring that consists of a polar boron-nitro­gen unit and a butadienyl moiety, making it an isoelectronic congener of benzene. Its strikingly similar geometry in conjunction with a significantly altered electron distribution has promoted research on mono- and polycyclic aromatic hydro­carbons (PAHs) with a BN substitution pattern. Several studies highlighted the BN-induced tailored adjustment of chemical, physical and optical properties, enabling the application of such heteroaromatics for instance as white-emitting layers in organic light-emitting diodes (Hoffmann *et al.*, 2021 ▸), as reversible hydrogen storage materials (Campbell *et al.*, 2010 ▸) or as building blocks in pharmaceuticals with increased bioavailability (Zhao *et al.*, 2017 ▸). Relatively few reports made use of the selectively deprotonable N*H* group (pK_a_ ≃ 24) to introduce electrophilic functional groups or metal atoms (Pan *et al.*, 2004 ▸; Lamm *et al.*, 2011 ▸; Baggett & Liu, 2017 ▸; Lindl *et al.*, 2023 ▸). 
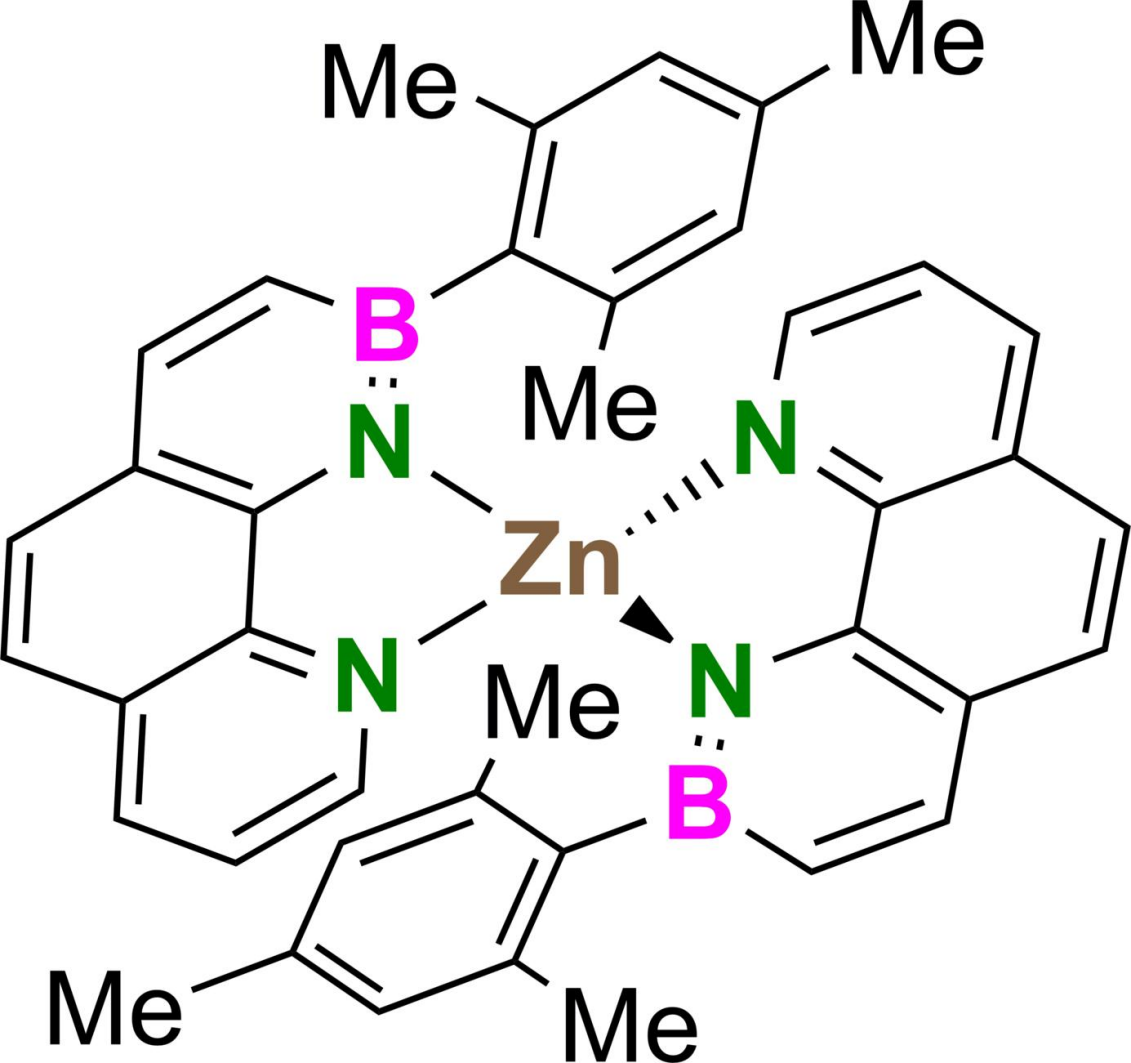


In a previous study (Appiarius *et al.*, 2021 ▸), a BN-substituted benzo[*h*]quinoline (**HL**), containing one 1,2-aza­borininyl- and one pyridyl subunit with both nitro­gen atoms beneficially preorganized for chelation was presented. In the context of this communication, we report on the synthesis and crystal structure of the 2:1 coordination complex of ligand **L** with zinc(II).

## 2. Structural commentary

**Figure 1:**
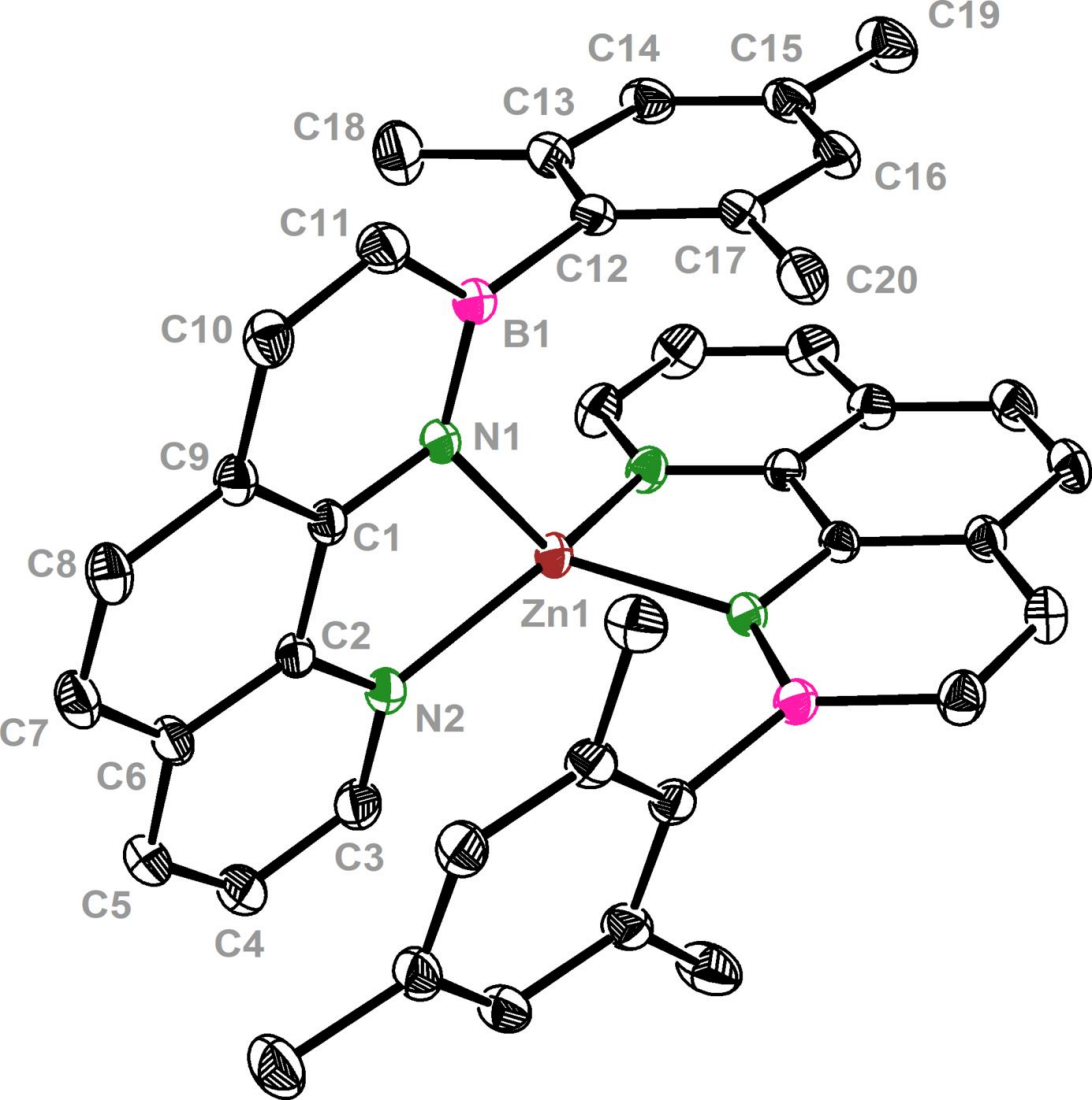
Mol­ecular structure of **ZnL_2_** with atom labeling. The image was generated with *ORTEP-3 for Windows* (Farrugia, 2012 ▸). Non-hydrogen atoms as displacement ellipsoids drawn at the 50% probability level. Hydrogen atoms were omitted for clarity. [Symmetry code: (i) −*x* + 1, *y*, –*z* + 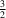 ].

The mol­ecular structure of the title compound (C_40_H_36_B_2_N_4_Zn, **ZnL_2_**) is illustrated in Fig. 1 ▸. The coordination complex crystallizes in the monoclinic *C*2/*c* centrosymmetric space group with one zinc(II) cation and one ligand mol­ecule in the asymmetric unit, being completed by the application of inversion symmetry at Zn^II^. The latter is fourfold coordinated by two types of N donors, namely the aza­borinine and pyridine subunits comprised in the BN-benzo[*h*]quinoline. This results in a significantly distorted tetra­hedral configuration [bond angle N1—Zn1—N2 84.72 (4)°; all other N—Zn—N bond angles > 118°, see Table 1 ▸]. The bond lengths within the 1,2-aza­borinine motif of the ligand [B1—N1: 1.4245 (17) Å, B1—C11: 1.5315 (19) Å, N1—C1: 1.3580 (14) Å] are in characteristic ranges (Paetzold *et al.*, 2004 ▸; Pan *et al.*, 2009 ▸), confirming electron delocalization and an elevated aromatic character. The N—Zn bond lengths [N1—Zn1: 1.9606 (10) Å, N2—Zn1: 2.0527 (10) Å] are in excellent agreement with bis­(2-(2′-pyrid­yl)pyrrol­yl)zinc [N_pyrrole_–Zn 1.9513 (18) Å, N_pyridine_–Zn 2.0444 (18) Å; Wang *et al.*, 2009 ▸], supporting the electronic similarities of 1,2-aza­borinine and pyrrole (Davies *et al.*, 2017 ▸). This contrasts with zinc complexes involving the geometrically similar but uncharged 1,10-phenanthroline ligand (N_pyridine_–Zn 2.13–2.20 Å) with higher coordination numbers of the central ion. All aromatic rings within the BN-PAH ligand are close to planar, with an average torsion angle of 2.2° and a maximum deviation of an atom from the mean aromatic plane of 0.0217 (8) Å. In contrast, the Zn^II^ ion is located 0.365 (2) Å out of the mean N1–C1–C2–N2 plane and points in the direction of the mesityl ring of the second ligand unit. The 1,2-aza­borinine motif and the attached planar mesityl group [maximum deviation from the mean aromatic plane: 0.0125 (9) Å] are oriented almost perpendicularly to each other, with an angle between their mean planes of 79.41 (4)°.

## 3. Supra­molecular features and Hirshfeld surface analysis

**Figure 2:**
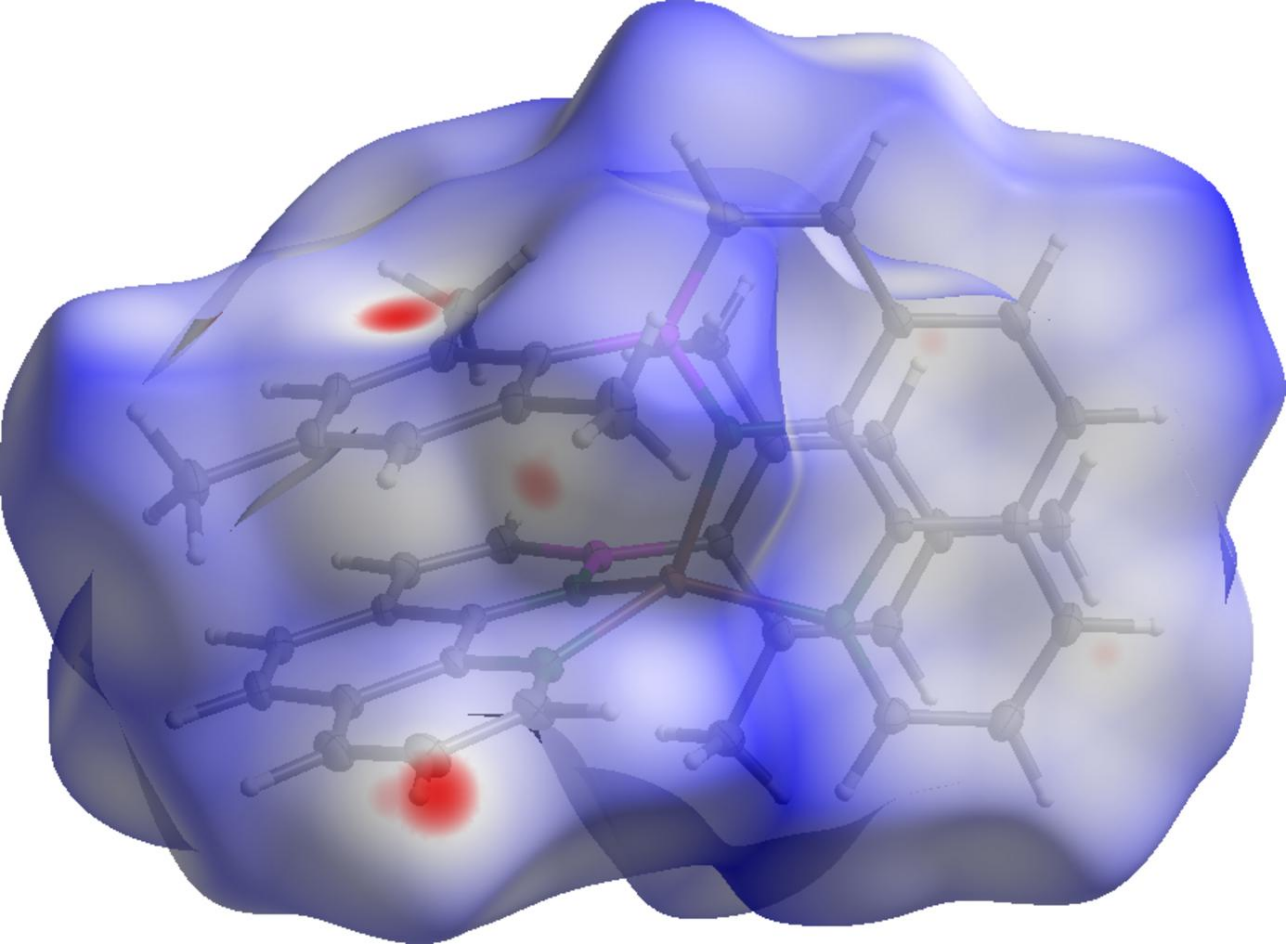
Hirshfeld surface of **ZnL_2_**, mapped over *d*_norm_ in the range between −0.1418 (red) and +1.6402 (blue).

**Figure 3:**
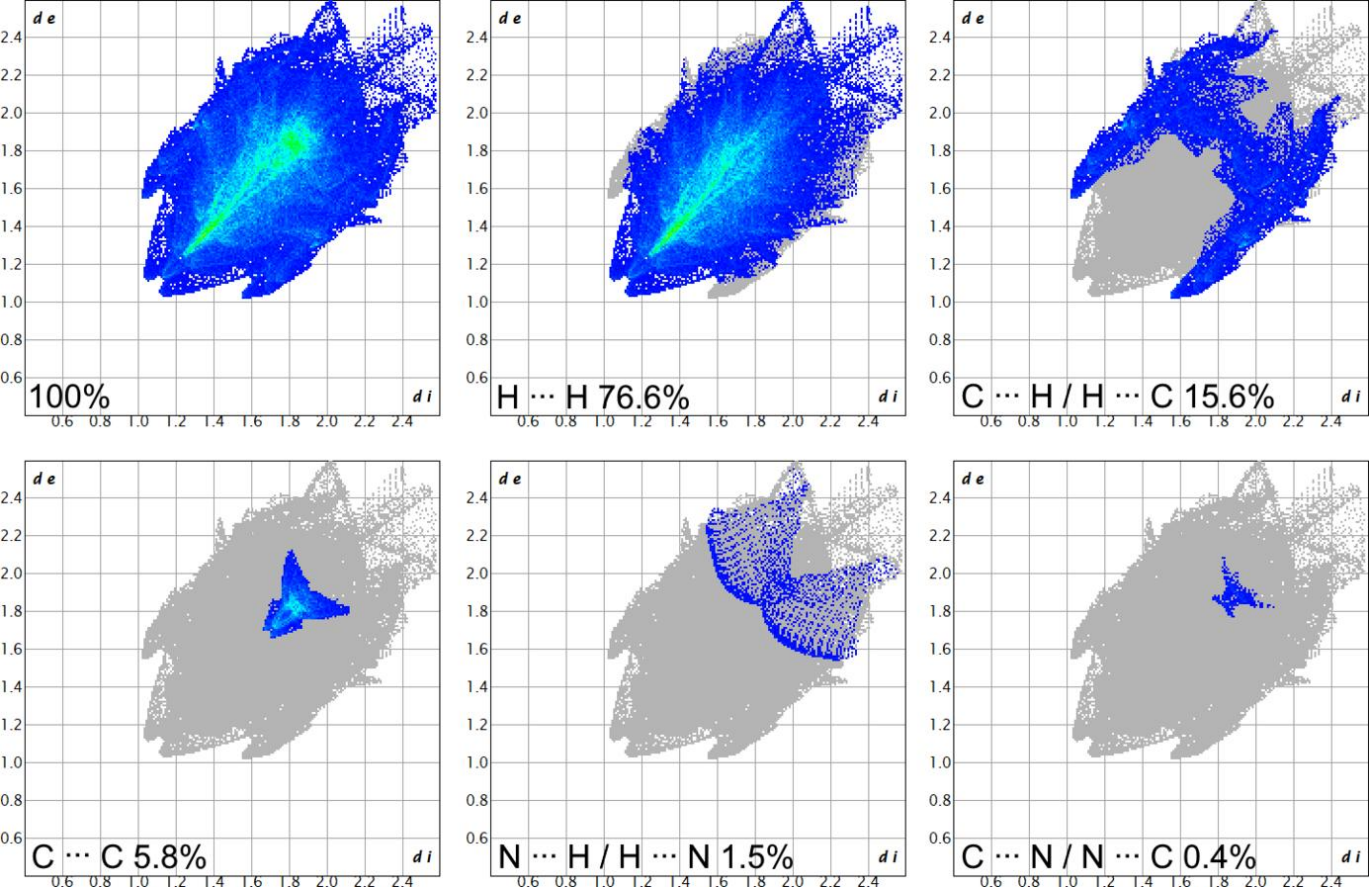
Two-dimensional fingerprint plots, showing the contributions of the individual elements in close atom contacts.

**Figure 4:**
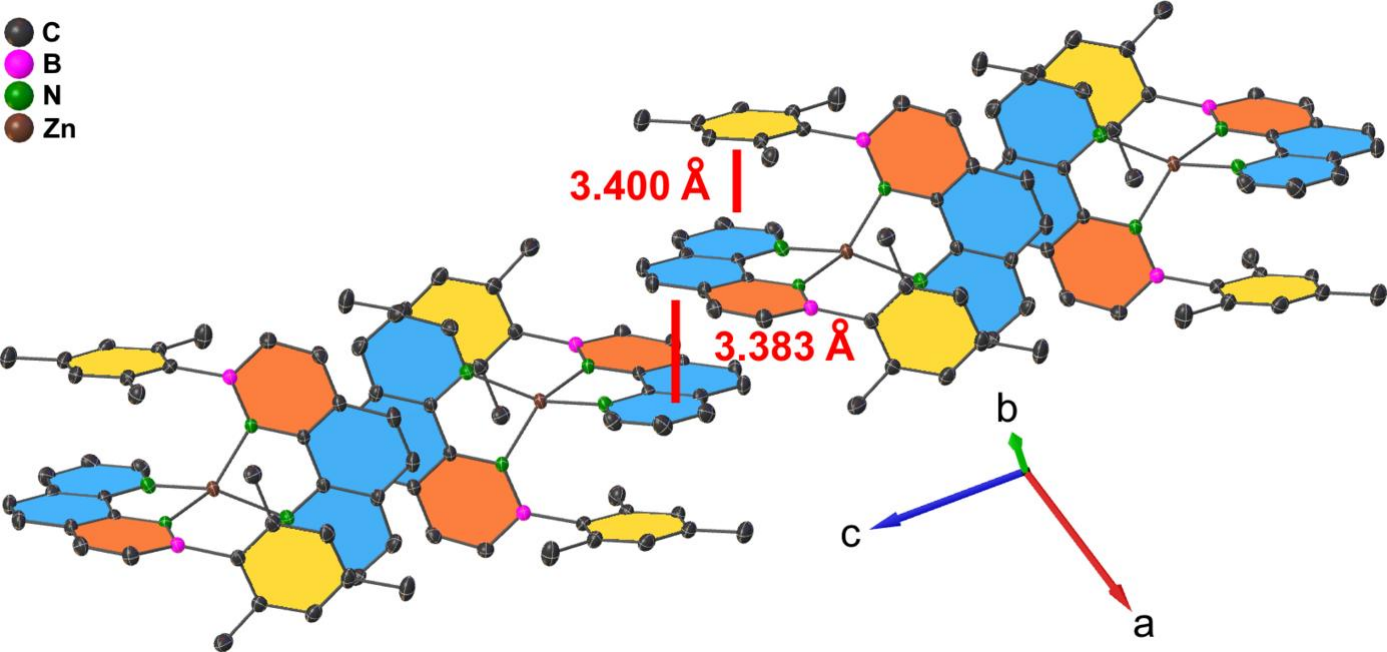
Section of the crystal packing, showing the π–π stacking inter­actions propagating in two dimensions. BN rings are shown in orange, mesityl rings in yellow, phenyl and pyridyl rings in blue.

A Hirshfeld surface (Hirshfeld, 1977 ▸, Fig. 2 ▸) and the respective two-dimensional fingerprint plots (Fig. 3 ▸) were generated using *CrystalExplorer21.5* (Spackman *et al.*, 2021 ▸) to analyze the inter­molecular inter­actions. No close atom contacts involving the boron and zinc heteroatoms and a negligible participation of the nitro­gen atom (N⋯H: 1.5%, C⋯N: 0.4%) were found. Therefore, the inter­molecular inter­actions were almost exclusively caused by van der Waals forces involving carbon and hydrogen. In particular, close H⋯H contacts and aromatic inter­actions dominate the overall inter­molecular inter­actions in a crystal. The ‘wings’ at the top left (*d*_i_ ≃ 1.05 Å and *d*_e_ ≃ 1.60 Å) and their pseudo-symmet­rical counterparts at the bottom right of the two-dimensional fingerprint plot correspond to C—H⋯π inter­actions. These are also mapped by several red spots on the Hirshfeld surface (Spackman & McKinnon, 2002 ▸). Moreover, considerable π–π stacking inter­actions are apparent by the light coloring of the Hirshfeld surface around the PAH backbone and intense C⋯C contacts (5.8%). The crystal packing shows that each aromatic ligand has one ligand unit of another mol­ecule in close proximity, so that pairs of almost parallel but slightly displaced sheets in two dimensions result (Fig. 4 ▸). In particular, the phenyl and pyridyl subunits of neighboring mol­ecules show a significant overlap, with an offset of only 1.181 (2) Å and a minimum inter­planar distance of 3.3826 (13) Å. On the other hand, the PAH scaffolds and the mesityl π-planes of the inverse ligand units are aligned almost coplanar [mesit­yl–pyridine inter­planar angle: 1.46 (5)°] with a similarly small minimum inter­planar distance [3.3996 (10) Å]. Therefore, intra­molecular π–π stacking contributes significantly to the overall stabilizing forces. We assume that the discussed, unusual off-plane position of the zinc ion and the increased angle between the mean mesityl plane and the B1—C12 bond [7.93 (8)°] also derives from this favorable stacking geometry.

## 4. Database survey

A survey of the Cambridge Structural Database (WebCSD version 1.9.32, accessed in July 2023 ▸; Groom *et al.*, 2016) revealed that 654 crystal structures of six-membered carbocycles with 1-aza-2-bora substitution patterns have been reported. Among these, 101 structures comprising *B*-mesityl substituents have been deposited, which involves aromatic 1,2-aza­borinine subunits for the most part. The crystal structures of 13 compounds with 1,2-aza­borinine substructures and nitro­gen–metal bonds have been published, of which different lithium solvates as well as potassium, beryllium, aluminum, gallium and tin complexes are included in one publication (Lindl *et al.*, 2023 ▸). Moreover, one study describes several complexes of a bidentate ligand with aluminum (Appiarius *et al.*, 2023 ▸). However, there are only three reports of 1,2-aza­borinines with N–transition-metal bonds, including zirconium (refcode JIZQEP; Pan *et al.*, 2008 ▸), ruthenium (refcode DOXBEY; Pan *et al.*, 2008 ▸) and iridium (refcode NEZXAV; Baschieri *et al.*, 2023 ▸). Also, the structure of a 6-pyridyl-1,2-aza­borinine has been reported, which is structurally similar to **HL** and was used for the preparation of a dimesitylboron complex (refcode WUGMIW; Baggett *et al.*, 2015 ▸). The search query for coordination complexes of zinc with 1,2-aza­borinine ligands did not yield any results.

## 5. Synthesis and crystallization

**Figure 5:**
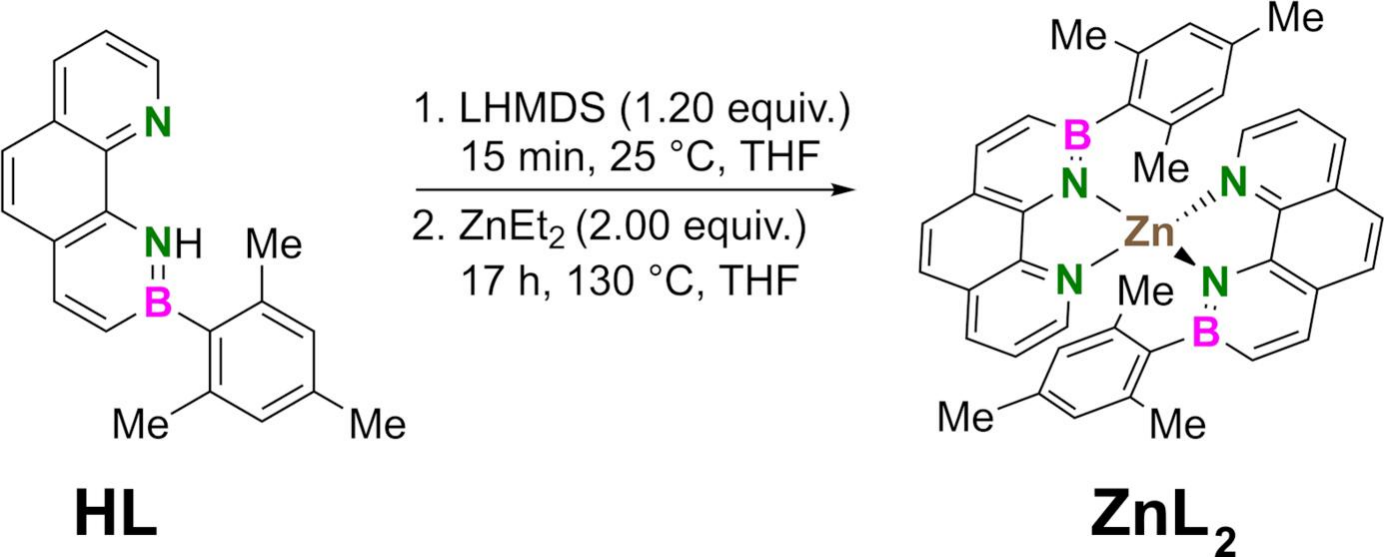
Reaction scheme for the synthesis of **ZnL_2_**.

The synthesis of **ZnL_2_** is shown in Fig. 5 ▸. Under argon at 298 K, 9,10-di­hydro-9-mesityl-10-aza-9-borabenzo[*h*]quinoline (**HL**, 29.8 mg, 100 µmol, 1.00 equiv., prepared according to Appiarius *et al.*, 2021 ▸) was dissolved in THF (1.5 mL). A solution of lithium bis­(tri­methyl­sil­yl)amide (1.0 *M* in THF, 120 µL, 1.20 equiv.) was added, before a solution of di­ethyl­zinc (15% *w*/*w* in hexa­nes, 230 µL, 2.00 equiv.) was added *via* a syringe. The mixture was heated to 428 K for 17 h while stirring. In a glove box, the volatiles were removed under reduced pressure. The residue was extracted with *n*-hexane (3 × 2 mL) and the solvent was removed. The crude product was dissolved in THF (500 µL) and *n*-hexane was allowed to diffuse into this solution over the course of 3 d. The light-yellow product (6.4 mg, 19%) was obtained as air-sensitive crystals suitable for X-ray diffraction analysis by repeating this process twice. **^1^H NMR** (600 MHz, THF-*d*_8_): δ = 8.37–8.32 (*m*, 4H, C3-*H* + C5-*H*), 8.07 (*d*, ^3^ *J* = 11.0 Hz, 2H, C10-*H*), 7.79 (*d*, ^3^ *J* = 8.7 z, 2H, C8-*H*), 7.38–7.35 (*m*, 2H, C4-*H*), 7.33 (*d*, ^3^ *J* = 8.7 Hz, 2H, C7-*H*), 6.90 (*d*, ^3^ *J* = 11.0 Hz, 2H, C11-*H*), 6.04 (*s*, 2H, C14-*H*), 5.61 (*s*, 2H, C16-*H*), 1.83 (*s*, 6H, C19-*H*), 1.79 (*s*, 6H, C18-*H*), 1.40 (*s*, 6H, C20-*H*) ppm. **^13^C{^1^H} NMR** (151 MHz, THF-*d*_8_): δ = 147.3 (C3), 145.8 (*C*1), 143.3 (*C*2), 143.0 (*C*10), 139.2 (*C*17), 139.2 (*C*5), 139.1 (*C*13), 134.8 (*C*15), 134.5 (*C*11), 131.2 (*C*8), 129.0 (*C*6), 127.1 (*C*14), 126.4 (*C*16), 126.1 (*C*9), 122.3 (*C*4), 116.1 (*C*7), 24.0 (*C*18), 23.8 (*C*20), 21.3 (*C*19) ppm. **^11^B{^1^H} NMR** (193 MHz, THF-*d*_8_): δ = 38.6 ppm. **MS** (EI): *m*/*z* 658.3 (3%) [**ZnL_2_**]^+^, 298.2 (100%) [HL]^+^. **HR-MS** (EI): *m*/*z* calculated for C_40_H_36_B_2_N_4_Zn^+^ 658.24259, found 658.24253 (Dev.: 0.06 mu, 0.09 ppm). **UV/Vis**: λ_abs_ = 296, 343, 358 nm. **Fluorescence**: λ_fl_ = 488 nm (λ_exc_ = 350 nm). Further experimental details can be found in the Supporting Information.

## 6. Refinement

Crystal data, data collection and structure refinement details are summarized in Table 2 ▸. Hydrogen atoms were positioned geometrically and refined using a riding model with C—H bond lengths of 0.95 Å (C—H) or 0.98 Å (C—H_3_). Isotropic displacement parameters (*U*_iso_) of these H atoms were fixed to 1.2 (C—H) or 1.5 (C—H_3_) of the values of the parent carbon atoms. Idealized methyl groups (C18—H_3_, C19—H_3_, C20—H_3_) were allowed to rotate.

## Supplementary Material

Crystal structure: contains datablock(s) I. DOI: 10.1107/S2056989023009192/dj2071sup1.cif

Structure factors: contains datablock(s) I. DOI: 10.1107/S2056989023009192/dj2071Isup2.hkl

Experimental details, unit cell packing views, NMR spectra, UV vis and fluorescence spectra. DOI: 10.1107/S2056989023009192/dj2071sup4.pdf

CCDC reference: 2302109

Additional supporting information:  crystallographic information; 3D view; checkCIF report

## Figures and Tables

**Table 1:** Selected geometric parameters (Å, °)

Zn1—N1	1.9606 (10)	N2—C2	1.3660 (16)
Zn1—N2	2.0527 (10)	N2—C3	1.3316 (17)
N1—C1	1.3580 (14)	C12—B1	1.5896 (18)
N1—B1	1.4245 (17)	C11—B1	1.5315 (19)
			
N1^i^—Zn1—N1	118.68 (6)	N1—Zn1—N2	84.72 (4)
N1^i^—Zn1—N2	122.48 (4)	N2—Zn1—N2^i^	128.26 (6)
			
Zn1—N1—C1—C9	166.76 (9)	Zn1—N1—B1—C12	20.10 (16)
Zn1—N1—C1—C2	−14.38 (13)	Zn1—N1—B1—C11	−163.25 (9)

Symmetry code: (i) 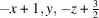 .

**Table 2:** Experimental details

Crystal data	
Chemical formula	[Zn(C_20_H_18_BN_2_)_2_]
*M*_r_	659.72
Crystal system, space group	Monoclinic, *C*2/*c*
Temperature (K)	100
*a*, *b*, *c* (Å)	20.0425 (14), 9.8589 (6), 17.1167 (9)
β (°)	105.172 (4)
*V* (Å^3^)	3264.3 (4)
*Z*	4
Radiation type	Mo *K*α
μ (mm^−1^)	0.79
Crystal size (mm)	0.24 × 0.12 × 0.09

Data collection	
Diffractometer	Bruker APEXII CCD
Absorption correction	Multi-scan (*SADABS*; Krause *et al.*, 2015 ▸)
*T*_min_, *T*_max_	0.677, 0.747
No. of measured, independent and observed [*I* > 2σ(*I*)] reflections	95271, 6577, 5466
*R*_int_	0.084
(sin θ/λ)_max_ (Å^−1^)	0.784

Refinement	
*R*[*F* ^2^ > 2σ(*F* ^2^)], *wR*(*F* ^2^), *S*	0.036, 0.097, 1.06
No. of reflections	6577
No. of parameters	216
H-atom treatment	H-atom parameters constrained
Δρ_max_, Δρ_min_ (e Å^−3^)	0.52, −0.40

Computer programs: *APEX2* and *SAINT* (Bruker, 2016 ▸), *SHELXT2018/2* (Sheldrick, 2015*a* ▸), *SHELXL2018/3* (Sheldrick, 2015*b* ▸), *OLEX2* (Dolomanov *et al.*, 2009 ▸), *ORTEP-3 for Windows* (Farrugia, 2012 ▸), and *publCIF* (Westrip, 2010 ▸).

## supplementary crystallographic information

### Crystal data

**Table:**

[Zn(C_20_H_18_BN_2_)_2_]	*F*(000) = 1376
*M**_r_* = 659.72	*D*_x_ = 1.342 Mg m^−^^3^
Monoclinic, *C*2/*c*	Mo *K*α radiation, λ = 0.71073 Å
*a* = 20.0425 (14) Å	Cell parameters from 9897 reflections
*b* = 9.8589 (6) Å	θ = 2.8–33.4°
*c* = 17.1167 (9) Å	µ = 0.79 mm^−^^1^
β = 105.172 (4)°	*T* = 100 K
*V* = 3264.3 (4) Å^3^	Irregular, light yellow
*Z* = 4	0.24 × 0.12 × 0.09 mm

### Data collection

**Table:**

Bruker APEXII CCD diffractometer	5466 reflections with *I* > 2σ(*I*)
φ and ω scans	*R*_int_ = 0.084
Absorption correction: multi-scan (SADABS; Krause *et al.*, 2015)	θ_max_ = 33.9°, θ_min_ = 2.8°
*T*_min_ = 0.677, *T*_max_ = 0.747	*h* = −31→31
95271 measured reflections	*k* = −15→15
6577 independent reflections	*l* = −26→26

### Refinement

**Table:**

Refinement on *F*^2^	Primary atom site location: dual
Least-squares matrix: full	Hydrogen site location: inferred from neighbouring sites
*R*[*F*^2^ > 2σ(*F*^2^)] = 0.036	H-atom parameters constrained
*wR*(*F*^2^) = 0.097	*w* = 1/[σ^2^(*F*_o_^2^) + (0.0416*P*)^2^ + 3.4887*P*] where *P* = (*F*_o_^2^ + 2*F*_c_^2^)/3
*S* = 1.06	(Δ/σ)_max_ < 0.001
6577 reflections	Δρ_max_ = 0.52 e Å^−^^3^
216 parameters	Δρ_min_ = −0.40 e Å^−^^3^
0 restraints	

### Special details

**Table:**

Geometry. All esds (except the esd in the dihedral angle between two l.s. planes) are estimated using the full covariance matrix. The cell esds are taken into account individually in the estimation of esds in distances, angles and torsion angles; correlations between esds in cell parameters are only used when they are defined by crystal symmetry. An approximate (isotropic) treatment of cell esds is used for estimating esds involving l.s. planes.

### Fractional atomic coordinates and isotropic or equivalent isotropic displacement parameters (Å^2^)

**Table:**

	*x*	*y*	*z*	*U*_iso_*/*U*_eq_	
Zn1	0.500000	0.38978 (2)	0.750000	0.01617 (6)	
N1	0.45636 (5)	0.28837 (11)	0.65132 (6)	0.01565 (17)	
N2	0.55610 (6)	0.48063 (11)	0.67988 (6)	0.01854 (19)	
C16	0.33235 (7)	0.14365 (15)	0.83074 (8)	0.0230 (2)	
H16	0.341124	0.087836	0.877572	0.028*	
C9	0.48444 (6)	0.22124 (13)	0.52702 (7)	0.0178 (2)	
C1	0.49408 (6)	0.30237 (12)	0.59647 (7)	0.01542 (19)	
C2	0.54576 (6)	0.40771 (12)	0.60987 (7)	0.0164 (2)	
C8	0.52494 (7)	0.24688 (14)	0.47125 (8)	0.0227 (2)	
H8	0.519116	0.190173	0.425081	0.027*	
C10	0.43285 (7)	0.11731 (14)	0.51402 (8)	0.0217 (2)	
H10	0.427219	0.059047	0.468550	0.026*	
C7	0.57165 (7)	0.35008 (15)	0.48219 (8)	0.0232 (2)	
H7	0.596684	0.366128	0.443013	0.028*	
C6	0.58310 (7)	0.43410 (14)	0.55225 (8)	0.0199 (2)	
C12	0.35616 (6)	0.19967 (13)	0.70258 (7)	0.0181 (2)	
C15	0.28518 (7)	0.24956 (15)	0.82289 (8)	0.0237 (2)	
C17	0.36714 (6)	0.11728 (14)	0.77154 (8)	0.0198 (2)	
C11	0.39151 (7)	0.09962 (14)	0.56519 (8)	0.0219 (2)	
H11	0.356957	0.031157	0.554933	0.026*	
C20	0.41803 (7)	0.00090 (15)	0.78396 (9)	0.0250 (3)	
H20A	0.463242	0.031778	0.816536	0.038*	
H20B	0.422293	−0.031172	0.731288	0.038*	
H20C	0.401508	−0.073317	0.812099	0.038*	
C14	0.27287 (7)	0.32946 (15)	0.75349 (8)	0.0237 (2)	
H14	0.240393	0.401532	0.746658	0.028*	
C19	0.24812 (8)	0.27744 (19)	0.88737 (10)	0.0329 (3)	
H19A	0.204988	0.225380	0.875753	0.049*	
H19B	0.237646	0.374471	0.887957	0.049*	
H19C	0.277652	0.250591	0.940294	0.049*	
C13	0.30729 (6)	0.30568 (14)	0.69395 (8)	0.0212 (2)	
C4	0.64006 (8)	0.61577 (16)	0.63859 (9)	0.0272 (3)	
H4	0.672060	0.688821	0.649815	0.033*	
C3	0.60226 (7)	0.58083 (14)	0.69353 (8)	0.0240 (2)	
H3	0.609776	0.630540	0.742618	0.029*	
C18	0.29227 (8)	0.39485 (18)	0.61986 (10)	0.0322 (3)	
H18A	0.333702	0.447143	0.619199	0.048*	
H18B	0.254432	0.457120	0.621039	0.048*	
H18C	0.278912	0.338272	0.571181	0.048*	
C5	0.63028 (7)	0.54297 (15)	0.56807 (9)	0.0251 (3)	
H5	0.655364	0.566043	0.529928	0.030*	
B1	0.40171 (7)	0.19187 (14)	0.63937 (8)	0.0171 (2)	

### Atomic displacement parameters (Å^2^)

**Table:**

	*U*^11^	*U*^22^	*U*^33^	*U*^12^	*U*^13^	*U*^23^
Zn1	0.01813 (9)	0.01939 (10)	0.01154 (8)	0.000	0.00488 (6)	0.000
N1	0.0160 (4)	0.0186 (4)	0.0133 (4)	0.0000 (3)	0.0055 (3)	0.0000 (3)
N2	0.0198 (5)	0.0209 (5)	0.0147 (4)	−0.0022 (4)	0.0042 (4)	0.0009 (3)
C16	0.0220 (6)	0.0303 (7)	0.0188 (5)	−0.0055 (5)	0.0089 (4)	0.0010 (5)
C9	0.0203 (5)	0.0193 (5)	0.0149 (5)	0.0015 (4)	0.0066 (4)	−0.0002 (4)
C1	0.0162 (5)	0.0178 (5)	0.0129 (4)	0.0023 (4)	0.0050 (4)	0.0016 (4)
C2	0.0167 (5)	0.0190 (5)	0.0139 (4)	0.0009 (4)	0.0048 (4)	0.0022 (4)
C8	0.0288 (6)	0.0247 (6)	0.0177 (5)	0.0025 (5)	0.0115 (5)	−0.0008 (4)
C10	0.0274 (6)	0.0212 (6)	0.0171 (5)	−0.0016 (5)	0.0070 (4)	−0.0034 (4)
C7	0.0266 (6)	0.0275 (6)	0.0194 (5)	0.0018 (5)	0.0127 (5)	0.0021 (5)
C6	0.0202 (5)	0.0227 (5)	0.0187 (5)	0.0006 (4)	0.0084 (4)	0.0046 (4)
C12	0.0152 (5)	0.0226 (6)	0.0169 (5)	−0.0034 (4)	0.0049 (4)	−0.0004 (4)
C15	0.0207 (5)	0.0314 (7)	0.0217 (6)	−0.0064 (5)	0.0105 (5)	−0.0050 (5)
C17	0.0174 (5)	0.0237 (6)	0.0192 (5)	−0.0038 (4)	0.0064 (4)	0.0015 (4)
C11	0.0233 (6)	0.0226 (6)	0.0203 (5)	−0.0049 (4)	0.0063 (4)	−0.0026 (4)
C20	0.0254 (6)	0.0256 (6)	0.0258 (6)	0.0007 (5)	0.0098 (5)	0.0059 (5)
C14	0.0183 (5)	0.0282 (7)	0.0265 (6)	−0.0010 (5)	0.0094 (5)	−0.0030 (5)
C19	0.0312 (7)	0.0431 (9)	0.0307 (7)	−0.0054 (6)	0.0191 (6)	−0.0066 (6)
C13	0.0179 (5)	0.0260 (6)	0.0209 (5)	−0.0004 (4)	0.0073 (4)	0.0016 (4)
C4	0.0264 (6)	0.0291 (7)	0.0260 (6)	−0.0084 (5)	0.0068 (5)	0.0041 (5)
C3	0.0266 (6)	0.0245 (6)	0.0195 (5)	−0.0064 (5)	0.0037 (5)	0.0006 (4)
C18	0.0294 (7)	0.0395 (8)	0.0309 (7)	0.0123 (6)	0.0137 (6)	0.0122 (6)
C5	0.0238 (6)	0.0293 (7)	0.0244 (6)	−0.0036 (5)	0.0100 (5)	0.0059 (5)
B1	0.0167 (5)	0.0197 (6)	0.0152 (5)	−0.0004 (4)	0.0045 (4)	0.0004 (4)

### Geometric parameters (Å, º)

**Table:**

Zn1—N1^i^	1.9606 (10)	C12—C13	1.4134 (18)
Zn1—N1	1.9606 (10)	C12—B1	1.5896 (18)
Zn1—N2	2.0527 (10)	C15—C14	1.393 (2)
Zn1—N2^i^	2.0527 (10)	C15—C19	1.5080 (19)
N1—C1	1.3580 (14)	C17—C20	1.5127 (19)
N1—B1	1.4245 (17)	C11—H11	0.9500
N2—C2	1.3660 (16)	C11—B1	1.5315 (19)
N2—C3	1.3316 (17)	C20—H20A	0.9800
C16—H16	0.9500	C20—H20B	0.9800
C16—C15	1.391 (2)	C20—H20C	0.9800
C16—C17	1.3966 (18)	C14—H14	0.9500
C9—C1	1.4035 (16)	C14—C13	1.3920 (18)
C9—C8	1.4281 (17)	C19—H19A	0.9800
C9—C10	1.4310 (18)	C19—H19B	0.9800
C1—C2	1.4421 (17)	C19—H19C	0.9800
C2—C6	1.4095 (16)	C13—C18	1.507 (2)
C8—H8	0.9500	C4—H4	0.9500
C8—C7	1.362 (2)	C4—C3	1.3964 (19)
C10—H10	0.9500	C4—C5	1.374 (2)
C10—C11	1.3652 (18)	C3—H3	0.9500
C7—H7	0.9500	C18—H18A	0.9800
C7—C6	1.4257 (19)	C18—H18B	0.9800
C6—C5	1.4092 (19)	C18—H18C	0.9800
C12—C17	1.4020 (17)	C5—H5	0.9500
			
N1^i^—Zn1—N1	118.68 (6)	C16—C17—C12	120.32 (12)
N1^i^—Zn1—N2^i^	84.72 (4)	C16—C17—C20	119.04 (12)
N1^i^—Zn1—N2	122.48 (4)	C12—C17—C20	120.62 (11)
N1—Zn1—N2^i^	122.48 (4)	C10—C11—H11	120.5
N1—Zn1—N2	84.72 (4)	C10—C11—B1	118.97 (12)
N2—Zn1—N2^i^	128.26 (6)	B1—C11—H11	120.5
C1—N1—Zn1	109.89 (8)	C17—C20—H20A	109.5
C1—N1—B1	120.99 (10)	C17—C20—H20B	109.5
B1—N1—Zn1	128.11 (8)	C17—C20—H20C	109.5
C2—N2—Zn1	107.62 (8)	H20A—C20—H20B	109.5
C3—N2—Zn1	133.06 (9)	H20A—C20—H20C	109.5
C3—N2—C2	118.90 (11)	H20B—C20—H20C	109.5
C15—C16—H16	119.2	C15—C14—H14	119.4
C15—C16—C17	121.69 (12)	C13—C14—C15	121.22 (13)
C17—C16—H16	119.2	C13—C14—H14	119.4
C1—C9—C8	119.26 (11)	C15—C19—H19A	109.5
C1—C9—C10	118.26 (11)	C15—C19—H19B	109.5
C8—C9—C10	122.46 (11)	C15—C19—H19C	109.5
N1—C1—C9	123.33 (11)	H19A—C19—H19B	109.5
N1—C1—C2	118.03 (10)	H19A—C19—H19C	109.5
C9—C1—C2	118.63 (10)	H19B—C19—H19C	109.5
N2—C2—C1	117.15 (10)	C12—C13—C18	119.97 (11)
N2—C2—C6	122.03 (11)	C14—C13—C12	120.70 (12)
C6—C2—C1	120.82 (11)	C14—C13—C18	119.32 (12)
C9—C8—H8	119.0	C3—C4—H4	120.5
C7—C8—C9	122.07 (12)	C5—C4—H4	120.5
C7—C8—H8	119.0	C5—C4—C3	119.03 (13)
C9—C10—H10	119.1	N2—C3—C4	122.63 (13)
C11—C10—C9	121.79 (12)	N2—C3—H3	118.7
C11—C10—H10	119.1	C4—C3—H3	118.7
C8—C7—H7	119.9	C13—C18—H18A	109.5
C8—C7—C6	120.21 (11)	C13—C18—H18B	109.5
C6—C7—H7	119.9	C13—C18—H18C	109.5
C2—C6—C7	118.90 (12)	H18A—C18—H18B	109.5
C5—C6—C2	117.29 (12)	H18A—C18—H18C	109.5
C5—C6—C7	123.81 (12)	H18B—C18—H18C	109.5
C17—C12—C13	117.92 (11)	C6—C5—H5	120.0
C17—C12—B1	123.59 (11)	C4—C5—C6	120.08 (12)
C13—C12—B1	118.06 (11)	C4—C5—H5	120.0
C16—C15—C14	118.10 (12)	N1—B1—C12	115.27 (11)
C16—C15—C19	121.23 (13)	N1—B1—C11	116.51 (11)
C14—C15—C19	120.67 (14)	C11—B1—C12	128.12 (11)
			
Zn1—N1—C1—C9	166.76 (9)	C10—C9—C1—C2	−179.75 (11)
Zn1—N1—C1—C2	−14.38 (13)	C10—C9—C8—C7	176.87 (13)
Zn1—N1—B1—C12	20.10 (16)	C10—C11—B1—N1	−2.14 (19)
Zn1—N1—B1—C11	−163.25 (9)	C10—C11—B1—C12	174.00 (13)
Zn1—N2—C2—C1	7.93 (13)	C7—C6—C5—C4	−177.57 (14)
Zn1—N2—C2—C6	−172.71 (10)	C15—C16—C17—C12	1.2 (2)
Zn1—N2—C3—C4	172.48 (11)	C15—C16—C17—C20	179.74 (13)
N1—C1—C2—N2	4.08 (16)	C15—C14—C13—C12	−0.3 (2)
N1—C1—C2—C6	−175.29 (11)	C15—C14—C13—C18	180.00 (14)
N2—C2—C6—C7	177.35 (12)	C17—C16—C15—C14	0.4 (2)
N2—C2—C6—C5	−2.32 (19)	C17—C16—C15—C19	−179.75 (13)
C16—C15—C14—C13	−0.8 (2)	C17—C12—C13—C14	1.89 (19)
C9—C1—C2—N2	−177.01 (11)	C17—C12—C13—C18	−178.44 (13)
C9—C1—C2—C6	3.63 (17)	C17—C12—B1—N1	−96.44 (15)
C9—C8—C7—C6	2.0 (2)	C17—C12—B1—C11	87.37 (17)
C9—C10—C11—B1	−1.2 (2)	C19—C15—C14—C13	179.30 (13)
C1—N1—B1—C12	−172.61 (11)	C13—C12—C17—C16	−2.33 (18)
C1—N1—B1—C11	4.04 (17)	C13—C12—C17—C20	179.19 (12)
C1—C9—C8—C7	−1.7 (2)	C13—C12—B1—N1	75.93 (15)
C1—C9—C10—C11	2.81 (19)	C13—C12—B1—C11	−100.25 (16)
C1—C2—C6—C7	−3.32 (18)	C3—N2—C2—C1	−178.47 (11)
C1—C2—C6—C5	177.02 (12)	C3—N2—C2—C6	0.89 (18)
C2—N2—C3—C4	0.8 (2)	C3—C4—C5—C6	−0.5 (2)
C2—C6—C5—C4	2.1 (2)	C5—C4—C3—N2	−1.0 (2)
C8—C9—C1—N1	177.72 (11)	B1—N1—C1—C9	−2.63 (18)
C8—C9—C1—C2	−1.14 (17)	B1—N1—C1—C2	176.23 (11)
C8—C9—C10—C11	−175.75 (13)	B1—C12—C17—C16	170.06 (12)
C8—C7—C6—C2	0.5 (2)	B1—C12—C17—C20	−8.42 (19)
C8—C7—C6—C5	−179.86 (14)	B1—C12—C13—C14	−170.92 (12)
C10—C9—C1—N1	−0.89 (18)	B1—C12—C13—C18	8.75 (19)

Symmetry code: (i) −*x*+1, *y*, −*z*+3/2.
