# Supplementary material for: Synthesis and crystal structure of bis­(9-mesityl-9,10-di­hydro-10-aza-9-borabenzo[h]quinolinato-κ2 N 1,N 10)zinc(II)
Source: Acta Crystallogr E Crystallogr Commun. 2023 Oct 24;79(Pt 11):1063–6. doi: 10.1107/S2056989023009192 (PMC10626948; doi:10.1107/S2056989023009192)
Supplement: Supplementary file 3 [file e-79-01063-sup4.pdf]

Supporting Information

Synthesis and crystal structure of bis(9,10-dihydro-9-mesityl-10-aza-9-borabenzo[*h*]quinolinato-κ^2*N*^{1},*N*^{10})zinc(II)

Yannik Appiarius [1,2], Pim Puylaert [3] and Anne Staubitz [1,2,*]

[1] University of Bremen, Institute for Organic and Analytical Chemistry, 28359 Bremen, Germany.
[2] University of Bremen, MAPEX Center for Materials and Processes, 28359 Bremen, Germany.
[3] University of Bremen, Institute for Inorganic Chemistry and Crystallography, 28359 Bremen, Germany.
***** Correspondence: staubitz@uni-bremen.de.

Abstract: The title compound, $C_{40}H_{36}B_2N_4Zn$ (**ZnL₂**), is an overall uncharged chelate that consists of two units of an N*H*-deprotonated 10-aza-9-borabenzo[*h*]quinoline ligand (L) per Zn(II) center. It was synthesized in two steps by treating protonated ligand **HL** with lithium bis(trimethylsilyl)amide and further conversion with diethylzinc. Its asymmetric unit comprises one ZnL fragment; the molecule is completed by application of inversion symmetry at Zn. Due to the fourfold coordination with nitrogen atoms, the zinc(II) ion is located in a distorted tetrahedral environment. Besides the relatively short N–Zn bonds, **ZnL$_2$** is characterized by the significant protrusion of the central ion from the plane of the ligand backbone. The crystal structure is consolidated by intra- and intermolecular π–π stacking interactions, while the polarized B–N bond is barely involved in any close atom contacts.

1. Experimental Details

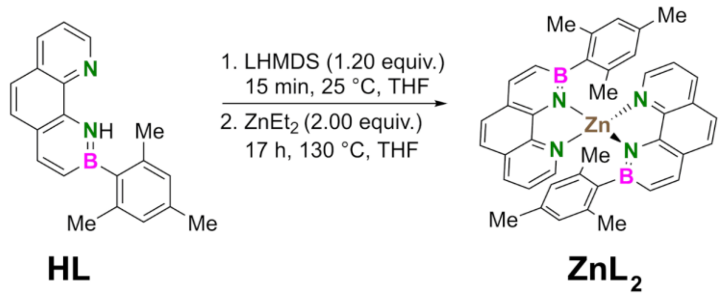

9,10-Dihydro-9-mesityl-10-aza-9-borabenzo[*h*]quinoline (**HL**) was prepared according to a reported procedure (Appiarius *et al.*, 2021). For the synthesis of **ZnL$_2$**, **HL** (29.8 mg, 100 µmol, 1.00 equiv.) was dissolved in THF (1.5 mL) in a pressure resistant reaction vial under an argon atmosphere at 298 K. Under stirring, a solution of lithium bis(trimethylsilyl)amide (Sigma Aldrich, 1.0 M in THF, 120 µL, 1.20 equiv.) was added *via* a syringe. A small aliquot was taken under inert conditions and by ^{1}H NMR spectroscopy, a full conversion into **LiL** was confirmed. **^{1}H NMR** (600 MHz, THF-d_8): δ = 8.60 (dd, ^{3}J = 4.4 Hz, ^{4}J = 1.7 Hz, 1H, C3-*H*), 8.20 (dd, ^{3}J = 8.0 Hz, ^{4}J = 1.7 Hz, 1H, C5-*H*), 7.97 (d, ^{3}J = 10.8 Hz, 1H, C10-*H*), 7.64 (d, ^{3}J = 8.4 Hz, 1H, C8-*H*), 7.38 (dd, ^{3}J = 8.0, 4.4 Hz, 1H, C4-*H*), 7.11 (d, ^{3}J = 8.4 Hz, 1H, C7-*H*), 6.80 (d, ^{3}J = 10.8 Hz, 1H, C11-*H*), 6.72 (s, 2H, C14-*H*, C16-*H*), 2.24 (s, 6H, C19-*H*), 2.15 (s, 12H, C18-*H*, C20-*H*) ppm. Under argon, a solution of diethylzinc (Alfa Aesar, 15% *w*/*w* in hexanes, 230 µL, 2.00 equiv.) was added to **LiL** *via* a syringe. While stirring, the mixture was heated to 428 K for 17 h. The reaction vessel was transferred into a nitrogen-filled glove box. The volatiles were removed under reduced pressure (up to 353 K, 5 mbar). The dark violet slurry was extracted with *n*-hexane (3 × 2 mL) and the solvents were removed. The crude product was dissolved in THF (500 µL) and *n*-hexane was allowed to diffuse into this solution over the course of 3 d to separate any remaining side-product, which remained at the bottom of the vial. The product was obtained as light yellow, air-sensitive crystals (6.4 mg, 19%) by repeating this process twice. These crystals were also suitable for structural analysis by X-ray diffraction and NMR spectroscopy. **^{1}H NMR** (600 MHz, THF-d_8): δ = 8.37 – 8.32 (m, 4H, C3-*H* + C5-*H*), 8.07 (d, ^{3}J = 11.0 Hz, 2H, C10-*H*), 7.79 (d, ^{3}J = 8.7 Hz, 2H, C8-*H*), 7.38 – 7.35 (m, 2H, C4-*H*), 7.33 (d, ^{3}J = 8.7 Hz, 2H, C7-*H*), 6.90 (d, ^{3}J = 11.0 Hz, 2H, C11-*H*), 6.04 (s, 2H, C14-*H*), 5.61 (s, 2H, C16-*H*), 1.83 (s, 6H, C19-*H*), 1.79 (s, 6H, C18-*H*), 1.40 (s, 6H, C20-*H*) ppm. **^{13}C{^{1}H} NMR** (151 MHz, THF-d_8): δ = 147.3 (C3), 145.8 (*C*1), 143.3 (*C*2), 143.0 (*C*10), 139.2 (*C*17), 139.2 (*C*5), 139.1 (*C*13), 134.8 (*C*15), 134.5 (*C*11), 131.2 (*C*8), 129.0 (*C*6), 127.1 (*C*14), 126.4 (*C*16), 126.1 (*C*9), 122.3 (*C*4), 116.1 (*C*7), 24.0 (*C*18), 23.8 (*C*20), 21.3 (*C*19) ppm. *C*12 was not detected due to its quaternary nature and the quadrupolar moment of the neighboring boron nucleus. **^{11}B{^{1}H} NMR** (193 MHz, THF-d_8): δ = 38.6 ppm. **MS** (EI, 70 eV, R ≈ 10 000): *m/z* 658.3 (3%) [ZnL$_2$]$^+$, 298.2 (100%) [HL]$^+$. **HR-MS** (EI, 70 eV, R ≈ 10 000): *m/z* calcd. for $C_{40}H_{36}B_2N_4Zn^+$ 658.24259, found 658.24253 (Dev.: 0.06 mu, 0.09 ppm). **UV/Vis**: λ_{abs} = 296, 343, 358 nm. **Fluorescence**: λ_{fl} = 488 nm (λ_{exc} = 350 nm). Solvents (THF, VWR, 99.7%; *n*-hexane, Honeywell, 97%) were dried and degassed with a solvent purification system, stored over activated molecular sieves (3 Å) and handled under inert conditions.

2. Crystallography

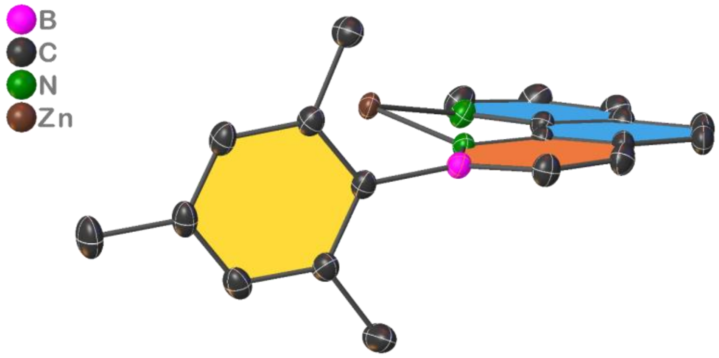

Figure S1. Asymmetric unit (ZnL), highlighting the off-plane position of the Zn(II) cation. BN-rings shown in orange, mesityl-rings in yellow, phenyl- and pyridyl-rings in blue.

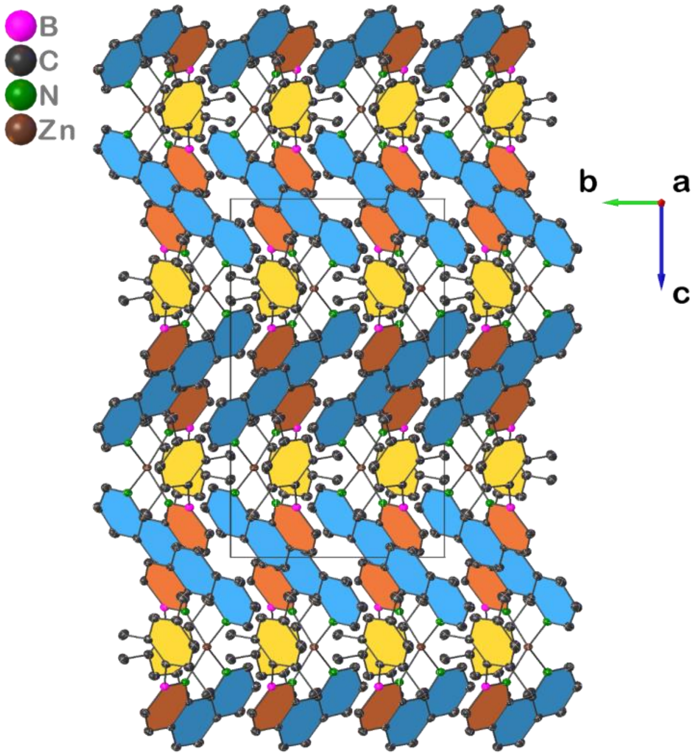

Figure S2. Unit cell packing view of a **ZnL_2** crystal along the *a*-axis. BN-rings shown in orange, mesityl-rings in yellow, phenyl- and pyridyl-rings in blue.

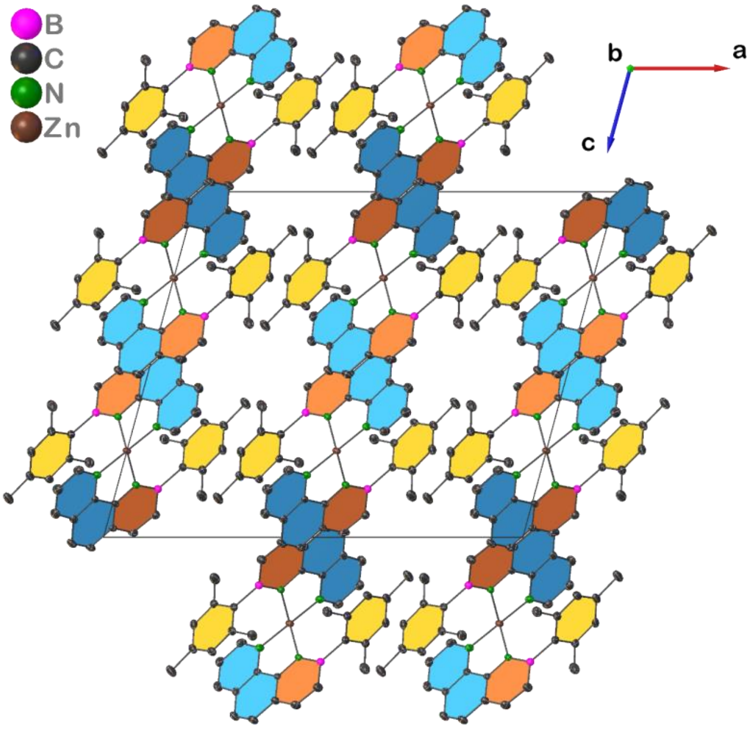

Figure S3. Unit cell packing view of a ZnL_2 crystal along the *b*-axis. BN-rings shown in orange, mesityl-rings in yellow, phenyl- and pyridyl-rings in blue.

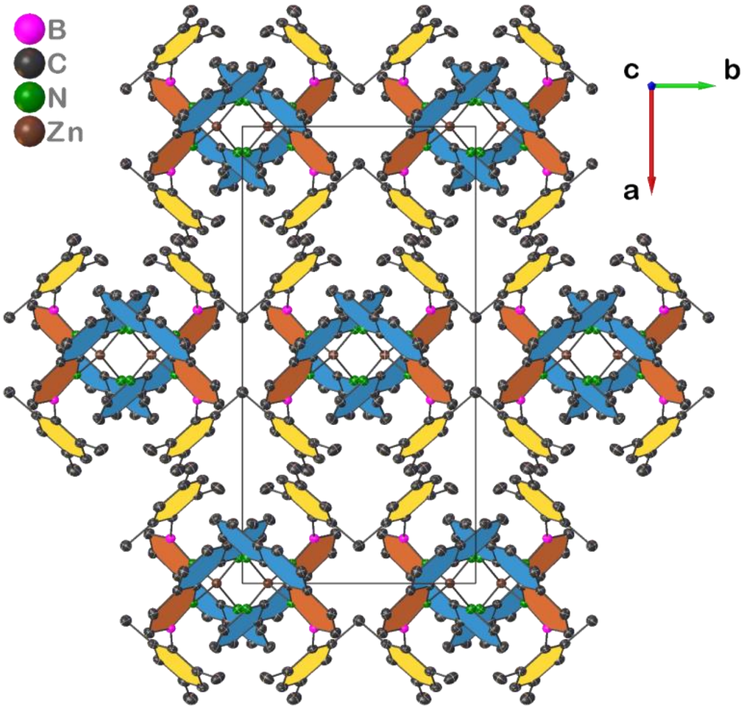

Figure S4. Unit cell packing view of a ZnL_2 crystal along the *c*-axis. BN-rings shown in orange, mesityl-rings in yellow, phenyl- and pyridyl-rings in blue.

3. NMR Spectra

^{1}H NMR (600 MHz, THF-d_8)

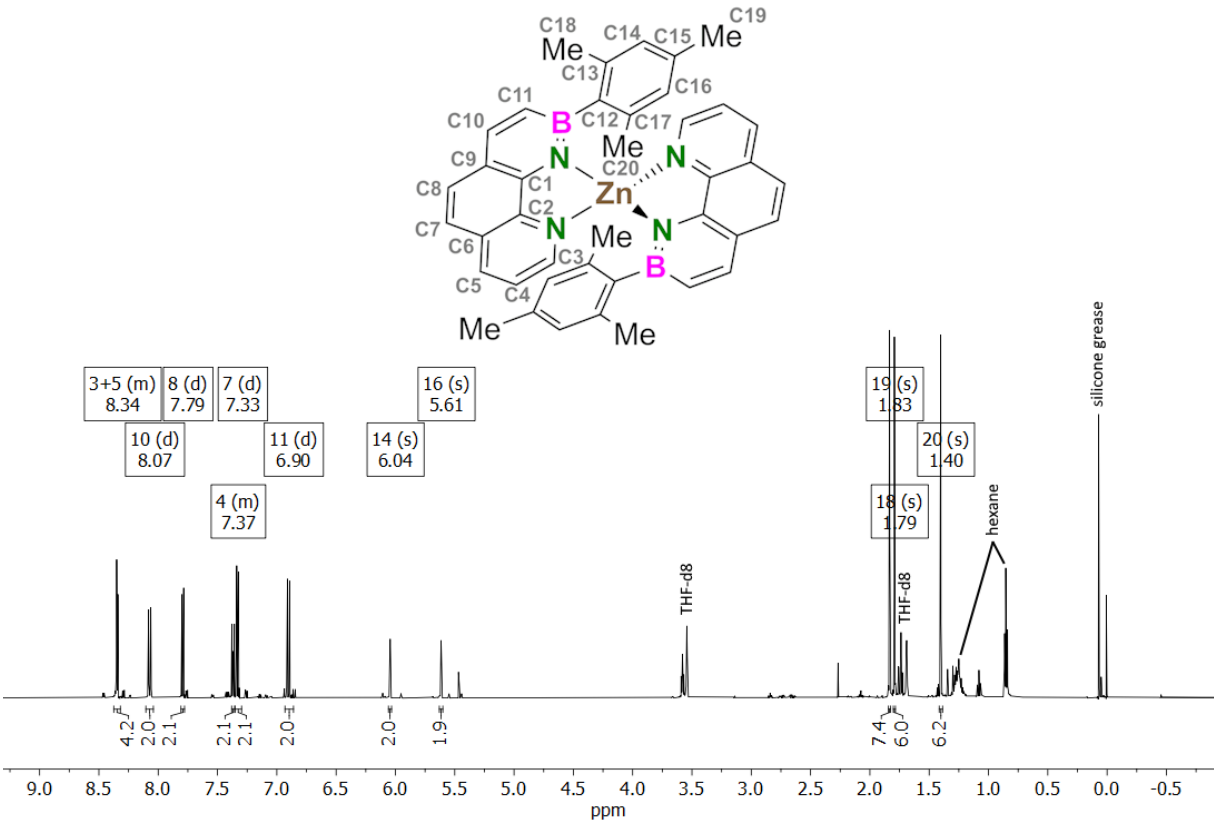

^{13}C{^{1}H} NMR (151 MHz, THF-d_8)

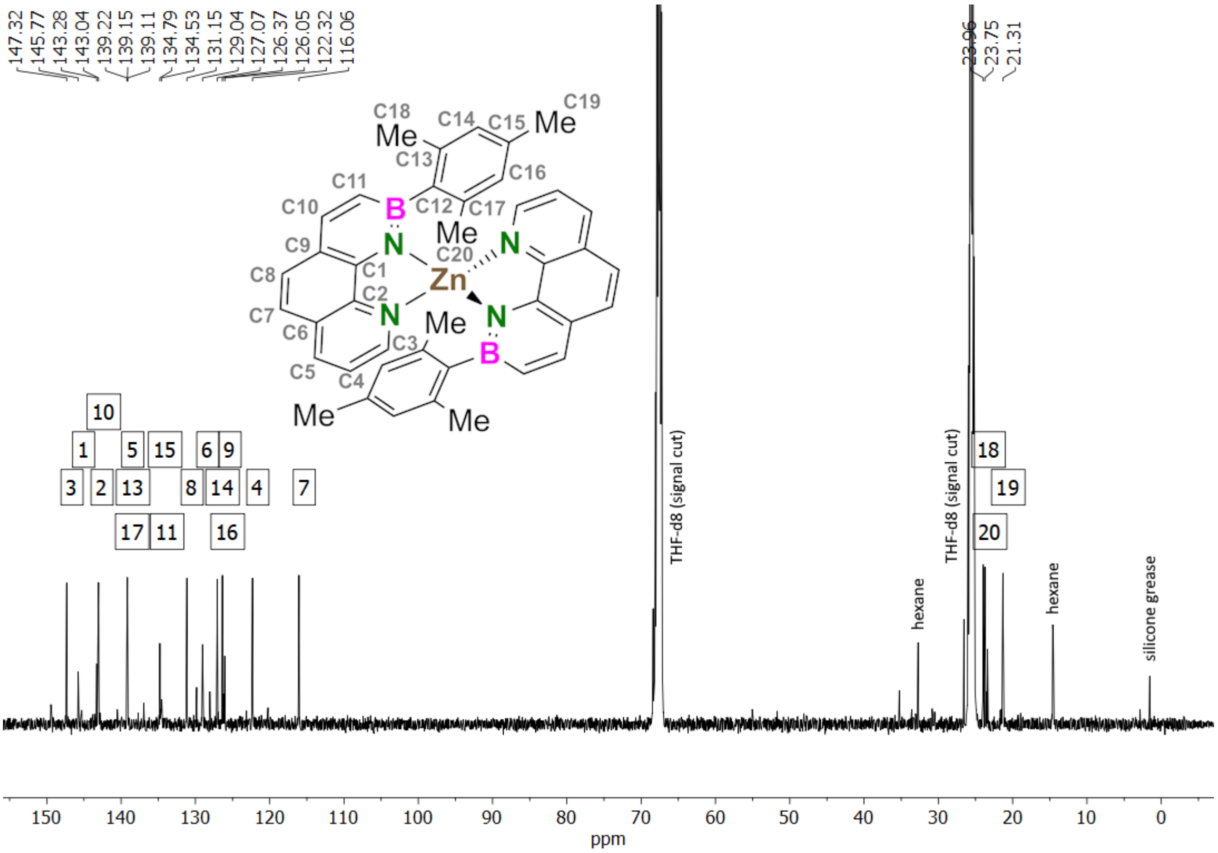

$^{11}B\{^1H\}$ NMR (193 MHz, THF-d_8)

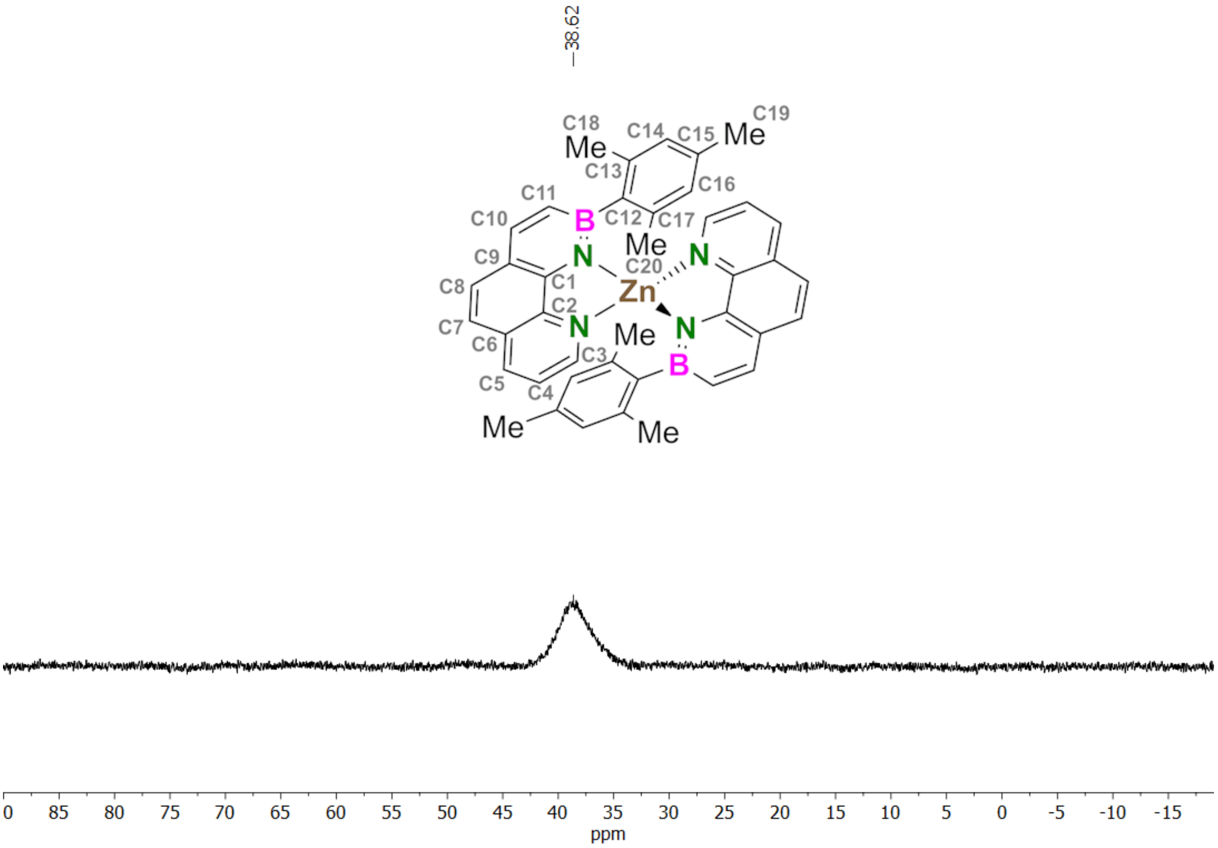

4. Optical Spectroscopy

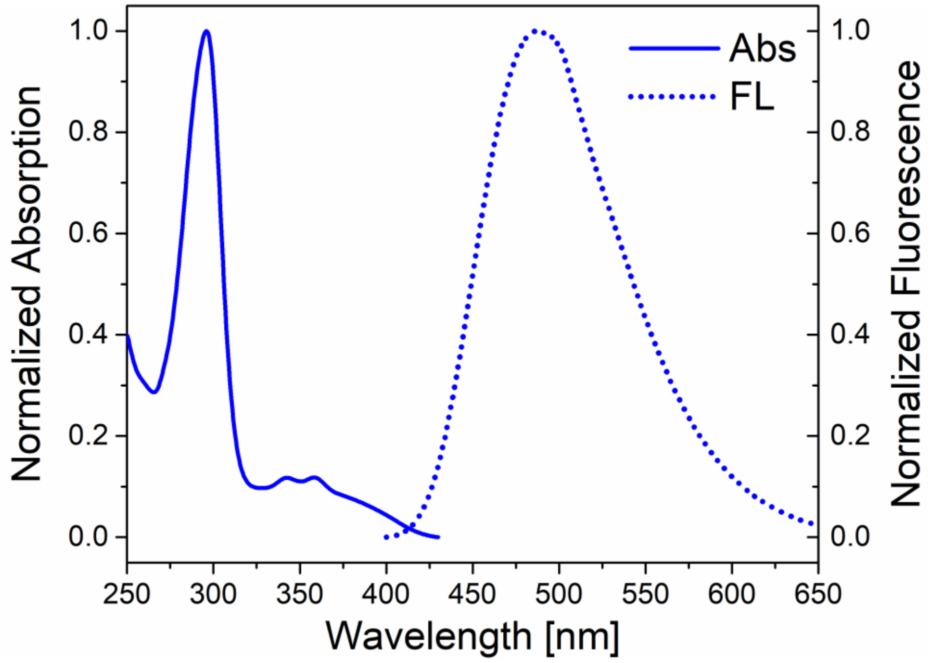

Figure S5. Normalized absorption (continuous) and fluorescence (dotted) spectra of ZnL_2.
